# Delmopinol hydrochloride reduces *Salmonella* on cantaloupe surfaces

**DOI:** 10.1002/fsn3.564

**Published:** 2017-12-15

**Authors:** Raúl O. Saucedo‐Alderete, Joseph D. Eifert, Renee R. Boyer, Robert C. Williams, Gregory E. Welbaum

**Affiliations:** ^1^ Department of Food Science and Technology Virginia Tech Blacksburg VA USA; ^2^ Department of Horticulture Blacksburg VA USA

**Keywords:** antimicrobial, cantaloupe, delmopinol hydrochloride, *Salmonella*

## Abstract

Since the surfaces of cantaloupes are highly rough or irregular, bacteria can easily attach and become difficult to remove. Appropriate postharvest washing and sanitizing procedures can help control *Salmonella* and other pathogens on cantaloupe or other melons during postharvest operations. Delmopinol hydrochloride (delmopinol) is a cationic surfactant that is effective for treating and preventing gingivitis and periodontitis. The application of delmopinol to two cantaloupe cultivars was evaluated for reducing the level of inoculated *Salmonella*. Athena and Hale's Best Jumbo (HBJ) cantaloupe rind plugs (2.5 cm. dia.) were inoculated with nalidixic acid‐resistant *Salmonella* Michigan (approx. 1.0 × 109 CFU/ml). After 15 min, rind plugs were sprayed with 10 ml of a delmopinol spray solution (0% or 1.0% vol/vol) and held at 35°C for 1 hr or 24 hr. Rind plugs were diluted with Butterfield's phosphate buffer, shaken and sonicated, and solutions were enumerated on 50 ppm nalidixic acid‐tryptic soy agar. The texture quality and color of additional cantaloupes were evaluated, after 1% delmopinol spray treatment, over 14‐day storage at 4°C. A 1.0% application of delmopinol after 1 hr reduced Salmonella concentration by ~3.1 log CFU/ml for both “HBJ” skin rind plugs and “Athena” stem scar rind plugs in comparison to the control (*p* < .05). No differences were observed in the texture and color (L*, a*, b* values) of 1% delmopinol‐treated cantaloupes as compared to control. Storage of cantaloupes treated with 1.0% delmopinol solution for 1 hr had a greater effect on reducing concentration of Salmonella compared to 24‐hr treatment. A surface spray application of 1% delmopinol on cantaloupes could be an alternative antimicrobial postharvest treatment that could make surface bacteria more susceptible to sanitizers or physical removal.

## 1 INTRODUCTION

As production and consumption of fresh fruits, including melons, and vegetables has increased in the United States (FDA 2001, Pollack, 2001), so has the importance of the microbiological safety of these products. Scientists are looking for new methods that increase the safety of produce while keeping the sensory qualities consumers expect in their fruits and vegetables. In the last few years, foodborne illness resulting from contamination of these raw agricultural commodities, particularly melons, has become an increasing concern (CDC 2011, 2012, FDA 2003). Several food safety programs, such as Good Agricultural Practices (GAPs), have been implemented to reduce outbreaks in areas from particular production fields. GAPs are guidelines and not “mandatory” for any country or company that wants to export its commodities to the USA (FDA, 1998).

After harvest, melons, including cantaloupe, honeydew, and watermelon, are susceptible to microbial contamination from mechanical damage and equipment, transport, grading and sorting, cleaning, packing and cooling, during distribution or by the final consumer. *Salmonella* spp. are directly associated with the use of products of animal origin including organic fertilizers and contaminated irrigation water. Direct field packing greatly reduces the cross‐contamination potential, but it is not recommended in areas of high rainfalls; so centralized packaging facilities are another option. Centralized packaging facilities are vulnerable to rapid cross‐contamination from shared or poorly cleaned water tanks, multiple melons harvested from different fields, and the possibility of fruit damage due to the additional manipulation of product. Risk factors associated with contamination by *Salmonella* in outbreaks in the US and Canada that were linked to melon and watermelon consumption were wash water temperature, contaminated hydro‐cooler water, damaged rind, rind fungus rot, workers’ hands, and contaminated conveyor belts and equipment (EFSA BIOHAZ Panel 2014).

*Salmonella* bacteria may attach to rough surfaces and build biofilm complexes making them hard to remove using just chlorine and tap water (Donlan, 2002; Ukuku & Fett, 2004). Parnell, Harris, and Suslow (2005) determined the effects of sanitizer and hot water treatments on microbial populations on cantaloupe surfaces and determined whether prior decontamination of melons by sanitizer treatment affects vulnerability to recontamination by *Salmonella*. The pathogen was reduced on the rind of cantaloupe by 1.8 log CFU/melon after soaking for 60 s in 200 ppm total chlorine, which was significantly better than the 0.7 log CFU/melon achieved when soaking in water. For both water and chlorine treatments, scrubbing with a vegetable brush was shown to be significantly (0.9 log CFU/cantaloupe) more effective than soaking alone. When honeydew melons were soaked or scrubbed in water, reductions of 2.8 log CFU/melon or 4.6 log CFU/melon (four of five samples), respectively, were observed. However, when water treatments were used, the presence of *Salmonella*‐positive samples, at adjacent and remote sites, indicated that bacteria were spread from the inoculated site on the rind to uninoculated sites either through the rinse water (40–70 CFU/ml of *Salmonella*) or scrub brush (400–500 CFU/brush). When 200 ppm total chlorine was used, *Salmonella* could not be detected in the water or on the scrub brush (Parnell et al., 2005).

Since, this pathogen is the predominant microorganism responsible for national and international outbreaks associated with consumption of cantaloupe (Richards & Beuchat, 2005), a new sanitizing option with high lethality is needed for cantaloupe and cantaloupe contact surfaces. In cases where *Salmonella* contamination has occurred at primary production or processing, at best, only a 1–2 log unit reduction in *Salmonella* can be achieved in the final product through washing procedures (EFSA BIOHAZ Panel, 2014). Some chemicals which are approved and used for other food processes, food products, or oral hygiene products and which have antimicrobial and anti‐biofilm properties, could have new applications for fresh agricultural commodities that have not been investigated.

Delmopinol hydrochloride ((3‐(4‐propylheptyl)‐4‐morpholinethanol); molecular formula: C_16_H_33_NO_2_) is an antiseptic and oral hygiene compound that may be useful as an antimicrobial for food. Decapinol^®^ is the trade name of the first oral hygiene products that contained delmopinol and was made commercially available by Sinclair Pharmaceutical Limited, later renamed as Sinclair IS Pharma (London, United Kingdom). Decapinol^®^ was first marketed in some countries of the European Union and was approved as a medical device by the U.S. Food and Drug Administration in 2005 for use in oral hygiene products (FDA 2005a,b). Delmopinol hydrochloride was classified as a medical device because its effectiveness is due primarily to a physical interference with dental plaque and biofilm formation and adherence of oral bacteria to teeth. This approval was based on clinical studies which showed that an oral rinse with 0.2% delmopinol decreases gingivitis up to 60% compared to no treatment when used as instructed with recommended brushing and flossing. The delmopinol molecule is amphiphilic, which has a polar end and nonpolar end. It acts as a surfactant which may lower the viscosity of solutions as well as interfere with colloidal structure (Klinge, Matsson, Attström, Edwardsson, & Sjödin, 1996; Simonsson, Arnebrant, & Petersson, 1991).

Delmopinol used as a direct spray application on foods or food contact surfaces could reduce *Salmonella* contamination. This chemical could be especially useful on surfaces with a highly irregular texture, such as the netted surface of cantaloupe, where a biofilm may be difficult to disrupt or remove. Short‐term tests with delmopinol have demonstrated little or no change in salivary bacterial counts, but significant decreases in the surface area covered with bacterial deposits (Hancock & Newell, 2001; Sjödin, Håkansson, Sparre, Ekman, & Aström, 2011). Theoretically, delmopinol could enable removal of exposed or hidden bacterial colonies, and cover a treated surface for several hours, repelling or reducing bacterial attachment (Hase, Attstrom, Edwardsson, Kelty, & Kisch, 1998; Yeung et al., 1995; Zee, Rundegren, & Attström, 1997).

Previous researchers have documented the inability of a variety of sanitizers and other treatments to completely remove and/or inactivate *Salmonella* inoculated onto cantaloupes (Sapers, 2001; Ukuku & Sapers, 2001). However, most of the research done so far have been aimed to replace treatments previously implemented (Alvarado‐Casillas, Ibarra‐Sánchez, Rodríguez‐ García, Martínez‐Gonzales, & Castillo, 2007; Ukuku, 2006; Ukuku & Fett, 2004) and not as additional steps or treatments for an extra food safety protocol. An additional step or postharvest technique should be available to cantaloupe packers and distributors to reduce the possibility of cross‐contamination by *Salmonella* and other pathogens.

The objectives of the study are to evaluate the efficiency of microbial reductions of *Salmonella*, by a postharvest treatment with delmopinol, on the complex netted surface of two cantaloupes “Athena” and “Hale's Best Jumbo” (“HBJ”). Additionally, this study evaluated the color and firmness of cantaloupe during refrigerated storage for up to 14 days at 4°C after a postharvest treatment with 1% delmopinol.

## 2 MATERIALS AND METHODS

### 2.1 Bacterial culture preparation

Difco™ Tryptic Soy Agar (TSA) (Becton–Dickinson and Company, Fisher Scientific, Pittsburgh, PA, USA) was diluted in 500 ml of distilled water, heated, dissolved and autoclaved at 121°C × 15 min and cooled. Then, 5 ml of 50 ppm nalidixic acid (Nal) stock solution was added and stirred for 10 min. Agar was poured into sterile petri dishes which were stored at room temperature to be used the next day.

For the nalidixic acid solution, sodium hydroxide solution (0.1 N NaOH) was prepared using 4 g of NaOH pellets (Certified ACS, Beat UN182, Fisher Chemicals, Fisher Scientific, Pittsburgh, PA, USA) in 1 liter of distilled water. Then, 0.5 g nalidixic acid (1‐Ethyl‐1,4‐Dihydro‐7‐methyl‐1,8‐naphthyridin‐4‐on‐3‐carboxylic acid, 99.5%) powder (Acros Organics, 99.5%, Lot A0272062) was added, and mixed slowly on a rotated magnetic plate. This solution (Nal stock) was sealed in a container, wrapped in aluminum foil and stored at 2–4°C for a maximum of 60 days.

*Salmonella enterica* Michigan, isolated from a cantaloupe illness outbreak, was obtained from Dr. Larry Beuchat at University of Georgia. A culture was made nalidixic acid‐resistant by consecutive transfers every 24 hr of isolated colonies from tryptic soy agar with increasing concentrations of nalidixic acid until colonies were resistant at a level of 50 ppm. Colonies were added to Tryptic Soy Broth (TSB) (Becton–Dickinson and Company, Fisher Scientific, Pittsburgh, PA, USA) tubes and incubated for 24 hr at 35°C ± 2°C. Positive cultures were transferred to vials and frozen in 80:20 glycerol solution at −75°C. Prior to each experiment, a culture vial was removed from frozen storage and defrosted slowly. A 0.1 ml aliquot of bacterial culture was added to 9.9 ml of TSB and incubated for 24 hr at 35°C ± 2°C. A sample randomly picked from each group was evaluated to check for viability in the presence of 50 ppm nalidixic acid. For each sample culture of *Salmonella* Michigan, 100 μl were plated on 40 ppm TSA+Nal plates, 50 ppm TSA+Nal plates, and 60 ppm TSA+Nal plates. Only colonies that grew on 50 ppm TSA+Nal plates were used in subsequent experiments. *Salmonella* identification was confirmed with a biochemical test kit (API 20 E, identification system for Enterobacteriaceae; bioMérieux, Inc., Durham, NC).

### 2.2 Cantaloupe harvest and selection

“Athena” and “Hale's Best Jumbo” cantaloupe were chosen because their surfaces are covered by a well‐developed, firm, deeply striated, and heavy netted skin and are more resistant to powdery mildew. The “Athena” is the most predominate commercial cantaloupe in the Eastern United States and “HBJ” is an heirloom melon that has been planted and sold in the Eastern U.S. for more than 100 years.

Cantaloupes were transplanted and direct seeded at the Virginia Tech College of Agriculture and Life Sciences farm facility (Kentland Farm) in Blacksburg, VA. First, seeds were planted at the greenhouse facility in 72 cell plug trays to obtain small melon transplants. These were transplanted in early June into black plastic mulch after the last frost. A second planting was done by direct seeding through holes into plastic mulch, to harvest the cantaloupes in sequential stages. Irrigation and fertilization was done using drip irrigation tubes under the plastic mulch. Plants were tended twice per week for weed removal, fruit rotation, and to confirm healthy growth. Insecticides were used only (under the Horticulture Department supervision) as a last resort and weeds were removed by hand. Cantaloupes were harvested when the stem part of the fruits was one‐third or one‐half off (slip stage), indicating that the fruits were ripe.

Undamaged cantaloupes were placed in a cleaned and sanitized plastic reusable box and transported to the Food Science and Technology building at Virginia Tech. Cantaloupes were sorted by size, cultivars, maturity, and cleanness. Over‐ripe, small and damaged cantaloupes were discarded, only whole good ones that did not show physical or insect damage or broken skins were used. Melons were transferred carefully to a clean water tank and debris was removed by hand and using a soft hair brush. Melons were rinsed using clean tap water and allowed to dry at room temperature (20°C–25°C) for 30 min. Cleaned and sorted melons were placed in dark plastic boxes and stored at 4°C for a maximum of 7 days in a walk‐in refrigerator.

### 2.3 Cantaloupe samples

Cantaloupes were transferred to a biological safety cabinet at room temperature (20°C) for 2 hr maximum before being sampled and treated. Cantaloupe rind plugs were collected (2.5 cm. diam., 2.5 cm. height, weight approx. 10 g) using a sanitized sterile cork bore plunger and the flesh adhering to the plug was trimmed off using a sterilized stainless steel single‐use scalpel. Rind plugs were inserted into a sterile sample container where 9.0 ml of Butterfield's phosphate buffer (3M, St. Paul, MN) was carefully added at the bottom of the container to prevent the sample from drying out and to preserve humidity.

Skin (SKN) samples were chosen that were well netted, thick, coarse, and corky, and stood out in bold relief over some part of the surface; the skin color (ground color) between the netting had changed from green to yellowish‐buff, yellowish‐gray, or pale yellow. Stem scar (SCR) samples were chosen that had a layer of cells around the stem that softens, yellowish cast rind, a smooth symmetrical, and shallow base dish‐shaped scar at the point of where the stem was attached. For each trial (3), 18 melons were used to obtain skin rind samples and 40 melons were used to obtain stem scar rings.

### 2.4 Delmopinol treatments

#### 2.4.1 Preparation of delmopinol solutions

Delmopinol hydrochloride powder, (Sinclair IS Pharma, London, United Kingdom) was mixed with distilled water to create a 1.0% (w/v) solution. Solutions were stored in clear airtight glass containers at room temperature, away from sunlight until further use, for a maximum of 60 days. Distilled water was used as a control (0% delmopinol). Two treatment applications were performed, (1) where *Salmonella* “Salm” was applied first, followed by a delmopinol solution (0 or 1.0%) spray “Del” treatment (Salm/Del), and (2) where the delmopinol solution (0 or 1.0%) spray treatment was applied first, followed by a *Salmonella* inoculation (Del/Salm).

#### 2.4.2 Bacteria and chemical spray application (Salm/Del)

Rind plugs were inoculated with 100 μl of a broth culture of *Salmonella* Michigan (approx. 1.0 × 10^9^ CFU/ml) using a sterile syringe. This broth culture of *Salmonella* was placed dropwise and spread evenly on the surface of the rind plugs. Then the melon rind plugs were left to stand for 1 hr or 24 hr, respectively, in an incubator at 35 ± 2°C. Then, plugs were sprayed; using a commercial bottle atomizer with a self‐adjusted spray nozzle, spraying at an angle of 45° to the surface of the rind plugs with 10 ml (3 pump sprays) of a delmopinol solution (0% or 1.0%) and left undisturbed for 15 min in a biosafety cabinet before microbiological analysis. For each of three replicated experiments per three trials, melon skin rind samples (3) and melon stem scar ring samples (3) were analyzed after 1‐hr storage, and melon skin rind samples (3) and melon stem scar ring samples (3) were analyzed after 24‐hr storage.

#### 2.4.3 Chemical spray and bacteria application (Del/Salm)

Rind plugs were sprayed using a commercial bottle atomizer with a self‐adjusted spray nozzle, spraying at an angle of 45° to the samples with 10 ml (3 pump sprays) of a delmopinol solution (0% or 1.0%) in a biosafety cabinet. After 15 min, rind plugs were inoculated with 100 μl of a broth culture of *Salmonella* Michigan (approx. 1.0 × 10^9^ CFU/ml) using a sterile syringe. The broth culture of *Salmonella* was placed dropwise and spread evenly on the surface of the rind plugs. Cantaloupe rind plugs were left to stand for 1 hr or 24 hr in an incubator at 35 ± 2°C. For each of the three replicated experiments per three trials, melon skin rind samples (3) and melon stem scar ring samples (3) were analyzed after 1‐hr storage, and melon skin rind samples (3) and melon stem scar ring samples (3) were analyzed after 24‐hr storage.

### 2.5 Microbiological analysis

Individual cantaloupe plugs were separately submerged in bottles of 90 ml of Butterfield's phosphate buffer. Bottles were shaken for 20 s by hand and decimal dilutions were plated on TSA‐Nal using an automated spiral plater (Autoplate 4000^®^ spiral plater; Spiral Biotech, Norwood, MA). Additionally, these plugs were transferred to a new cup with fresh Butterfield's phosphate buffer (99 ml) and sonicated at 75 joules (15 watts for 5 s) in three intervals (1:1:1) using a 130 Watt, 20 kHz, ultrasonic processor (Cole Palmer Instruments, Vernon Hills, IL, USA) with a 6 mm (1⁄4″) diameter ultrasonic probe. These sample dilutions were also plated on TSA‐Nal using an automated spiral plater (Autoplate 4000^®^ spiral plater; Spiral Biotech, Norwood, MA). Plates were held at 35 ± 2°C for 24 hr. Colonies were enumerated using a ProtoCOL^®^ automated colony counter (Microbiology International, Frederick, MD). All samples were plated in duplicate and the experiment was replicated three times. The recovered cell concentrations for each sample enumerated with and without sonication were summed together prior to additional calculations of mean recovery and statistical significance.

### 2.6 Color analysis

Fifteen whole cantaloupes (“Athena”) were sprayed using a bottle atomizer with a self‐adjusted spray nozzle, spraying at an angle of 45° to the cantaloupe with 40 ml (5 spray pumps) of a 0% or 1.0% delmopinol spray solution and stored at 4°C for 1, 2, 5, 7, and 14 days. Color measurements were recorded, for three replicate experiments, using a portable Minolta CR‐300 Chromameter (Konica Minolta, Ramsey, NJ, USA). For each sample, three readings were interpreted using the Hunter CIE L*a*b* (CIELAB) scale, where L* indicates the level of lightness and darkness, the a* value indicates the degree of redness and greenness, and the b* value indicates yellowness and blueness. A combination of these values, which represent an overall color change, were reported as Δ*E* (where, ΔE* = [ΔL*^2^ + Δa*^2^ + Δb*^2^]^1/2^). The instrument was standardized using black and white tiles previous to each reading, per the procedure described by the manufacturer of the Chromameter.

### 2.7 Texture analysis

Fifteen whole cantaloupes (“Athena”) were sprayed using a commercial bottle atomizer with self‐adjusted spray nozzle, spraying at an angle of 45° with 40 ml (5 spray pumps) of 0% or 1.0% delmopinol spray solution and stored at 4°C for 1, 2, 5, 7, and 14 days. These cantaloupes were not additionally tested for color or microbial recovery. The firmness of the cantaloupes was analyzed using a TA‐XT Plus, series 10545, texture analyzer (Texture Technologies, Scarsdale, New York, USA) with a model TA‐23 plunger (½” diameter, ¼ R end, 3″ tall). The auto trigger was used with 5 gm force and a 2.0 mm/s test distance penetration speed. Readings were collected in triplicate.

### 2.8 Statistical analysis

Three replicate experiments were conducted and two samples (skin rind plugs (SKN) or stem scar rind plugs (SCR)) of each treatment were analyzed for *Salmonella* Michigan at each sampling time. Data were analyzed by randomized complete block factorial design using the general linear model (GLM) procedure of Statistical Analysis Software, Version 9.13 (SAS Institute, Cary, NC). Significant differences (*p *≤* *.05) in microbial recovery due to delmopinol treatment, storage time (1 hr, 24 hr), and order of application (Del/Salm) or Salm/Del) were determined using Tukey′s multiple range test.

## 3 RESULTS AND DISCUSSION

**Figure 1:**
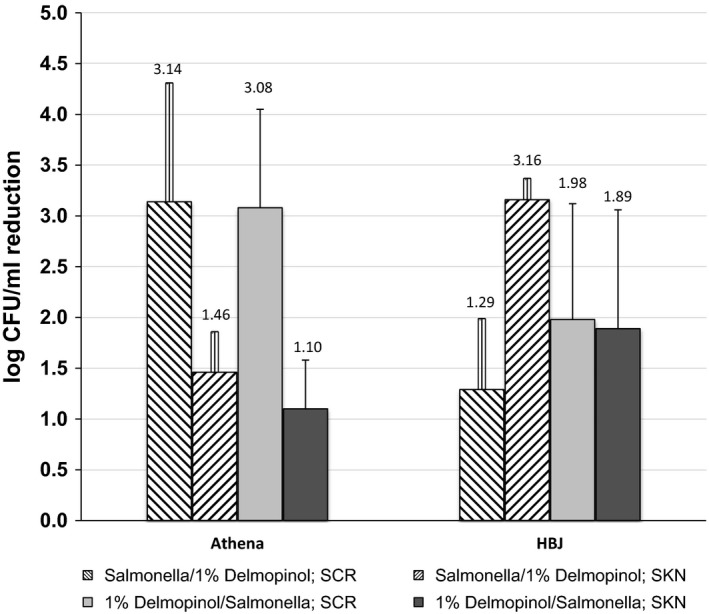
Log CFU/ml reduction in *Salmonella* from stem scar rind plugs (SCR) and skin rind plugs (SKN) from Athena and Hale Best Jumbo (HBJ) cantaloupe after 1‐hr incubation periods at 35°C, where 1% delmopinol was applied after or before *Salmonella*

Bacterial pathogens which may be present on the rind surface of cantaloupe may be reduced, but are unlikely to be eliminated by washing. Washing or immersing netted melons in water has the potential for pathogen internalization and cross‐contamination to other melons (Produce Marketing Association 2013). In this study, a 1.0% delmopinol direct spray solution was evaluated for reduction in *Salmonella* Michigan on skin rind plugs (SKN) and stem scar rind plugs (SCR) of two cantaloupe cultivars, Athena and Hale's Best Jumbo (HBJ). For the Athena cultivar cantaloupe, population reductions of *Salmonella* on stem scar rind plugs (SCR) was approximately 3.1 log CFU/ml, greater than the control group, when 1% delmopinol (Del) was applied either 1 hr before or after the *Salmonella* (Del/Salm or Salm/Del) (Table 1, Figure 1). *Salmonella* was reduced between 1.1 and 1.46 log CFU/ml on skin rind plugs (SKN). For both skin rind plugs (SKN) and stem rind plugs (SCR), *Salmonella* populations were significantly lower (*p *<* *.05) after 1 hr with each delmopinol treatment. *Salmonella* reduction (from control) after 24‐hr storage of SCR was <1 log CFU/ml, and for SKN reduction was <1 log CFU/ml when 1% delmopinol was applied after *Salmonella* (Table 1). Other researchers have reported differences between pathogen reduction on cantaloupes between stem scar areas and rind surfaces when antimicrobials are evaluated. For example, Webb, Davey, Erickson, and Doyle (2013) reported that 2% levulinic acid plus 0.2% sodium dodecyl sulfate was effective for reducing *Salmonella* on the netted rind surface of cantaloupes, but did not substantially reduce *Salmonella* on stem scar tissue.

**Table 1:** Log_10_ CFU/ml recovery of *Salmonella* from stem scar rind plugs (SCR) and skin rind plugs (SKN) of “Athena” cantaloupe after 1‐hr and 24‐hr incubation periods at 35°C and 1% delmopinol spray solution applied

Treatment	Stem scar plugs (SCR)		Skin plugs (SKN)	
Order of application	1 hr	24 hr	1 hr	24 hr
*Salmonella*, distilled water	8.12 ± 1.35^a^	9.58 ± 0.15^a^	7.51 ± 0.59^a^	9.85 ± 0.16^a^
*Salmonella*, 1% Delmopinol	4.98 ± 1.17^b^	9.45 ± 0.14^a^	6.05 ± 0.40^b^	8.93 ± 0.78^b^
1% Delmopinol^a^, *Salmonella*	5.04 ± 0.97^b^	9.02 ± 0.50^a^	6.41 ± 0.48^b^	8.24 ± 0.20^b^

Column means with the same letter are not significantly different (*p *<* *.05).

a Delmopinol hydrochloride (delmopinol) solution sprayed and left undisturbed for 15 min.

For Hale's Best Jumbo (HBJ)′s cultivar cantaloupe, population reductions of the *Salmonella* on skin rind plugs (SKN) was significantly greater (*p *<* *.05) when delmopinol was applied 1 hr after the bacteria (3.16 log CFU/ml) or 1 hr before the bacteria (reduction of 1.89 log CFU/ml greater than the control) (Table 2, Figure 1). Population reductions in the *Salmonella* on stem scar plugs (SCR) (1.98 log CFU/ml) was higher when 1% delmopinol was applied 1 hr before the bacteria (Del/Salm), but recovered *Salmonella* was not significantly lower from the control. *Salmonella* reduction (from control) after 24‐hr storage of skin rind plug (SKN) and stem scar rind plug (SCR) was <1 log CFU/ml (Table 2).

**Table 2:** Log CFU/ml recovery of *Salmonella* from stem scar rind plugs (SCR) and skin rind plugs (SKN) of “Hales Best Jumbo (HBJ)” cantaloupe after 1‐hr and 24‐hr incubation periods at 35°C and 1% delmopinol spray solution applied

Treatment	Stem scar plugs (SCR)		Skin plugs (SKN)	
Order of application	1 hr	24 hr	1 hr	24 hr
*Salmonella*, distilled water	7.34 ± 0.15^a^	8.71 ± 0.78^a^	7.44 ± 0.48^a^	9.33 ± 0.17^a^
*Salmonella*, 1% Delmopinol	6.05 ± 0.70^a^	7.95 ± 0.90^a^	4.28 ± 0.21^b^	8.54 ± 0.07^b^
1% Delmopinol^a^, *Salmonella*	5.36 ± 1.14^a^	8.30 ± 0.43^a^	5.55 ± 1.17^b^	8.73 ± 0.27^b^

Column means with the same letter are not significantly different (*p *<* *.05).

a Delmopinol hydrochloride (delmopinol) solution sprayed and left undisturbed for 15 min.

Simonsson, Hvid, Rundergreen, and Edwardsson (1991) and Rundergreen, Hvid, Johansson, and Aström (1992) suggested a small bactericidal effect of delmopinol HCl and the effect of dissolving formed plaque in the absence of mechanical plaque control, but the exact mode of action is not yet known (Eley, 1999; Hancock & Newell, 2001; Brandon‐Kelsch, 2011). However, Zee et al. (1997) and Burgemeister, Decker, Weiger, and Brecx (2001) research on planktonic and attached cells also showed a marked decrease in vitality following exposure to 0.2% delmopinol. They suggested that delmopinol does not just possess a bactericidal effect, but also possesses an anti‐aggregating effect rather than an anti‐adhesive effect on the pioneer bacteria. When there are existing plaque colonies, the cohesive forces between the bacteria are reduced by delmopinol, which makes removal by mechanical means much easier.

Experiments conducted to assess the relative strength of attachment of *Salmonella* on cantaloupe rinds demonstrated an increasing strength of attachment from day 0 to 7 during storage (Ukuku & Fett, 2002). Annous, Solomon, Cooke, and Burke (2005), and found that extracellular polymeric substance formation (biofilm) occurred rapidly following introduction of cells (2 hr at 20°C) onto the cantaloupe rind. Ukuku and Sapers (2001) speculated that increased contact time allowed for strong microbial attachment to the cantaloupe surface and the formation of a bacterial extracellular polymeric substance prior to sanitation. They found that *Salmonella* Michigan produces large amounts of these substances following their introduction onto the cantaloupe rind.

Both “Athena” and “HBJ” have well‐netted, thick, coarse, and corky skin, where pathogenic bacteria could hide and attach to many places on the surface. While *Salmonella* populations were reduced by the treatments, some organisms remained on cantaloupe sample surfaces. After 1 hr, the application of 1% delmopinol, applied before or after *Salmonella* (Del/Salm or Salm/Del) on “Athena” stem scar rind plugs resulted in a similar log reduction (~3.1 log CFU/ml) compared to the lesser reduction on skin rind plugs (~1.1–1.4 log CFU/ml less than control). This 1% delmopinol spray application on stem scar rind plugs (SCR) was more effective on “Athena” (~3.1 log CFU/ml) than “HBJ” where the log reduction was ~1.3–2.0 log CFU/ml. This difference could be attributed to “Athena” stem scars (SCR) which are smaller, less soft, and have less susceptibility to fracture than “HBJ” stem scars. Also, bacteria may not have had sufficient time to internalize into the cantaloupes or have direct contact with 1% delmopinol. On the other hand, treatment applications where *Salmonella* was applied first and then followed by a 1% delmopinol spray treatment (Salm/Del), the log reduction was significantly greater on “HBJ” skin rind plugs (SKN), after 1 hr, than on stem scar rind plugs (SCR) of “HBJ” and stem scar rind plugs (SCR) of “Athena”.

No significant difference in *Salmonella* recovery was observed on either “Athena” or “HBJ” cantaloupe after 24‐hr storage on both stem scar rind plugs (SCR) and skin rind plugs (SKN). Storage of cantaloupes treated with 1.0% delmopinol spray solution for 1 hr had a greater effect on reducing *Salmonella* compared to 24‐hr treatment for both “Athena” and “HBJ,” suggesting a rapid and short term bactericidal effect on bacteria cells.

**Figure 2:**
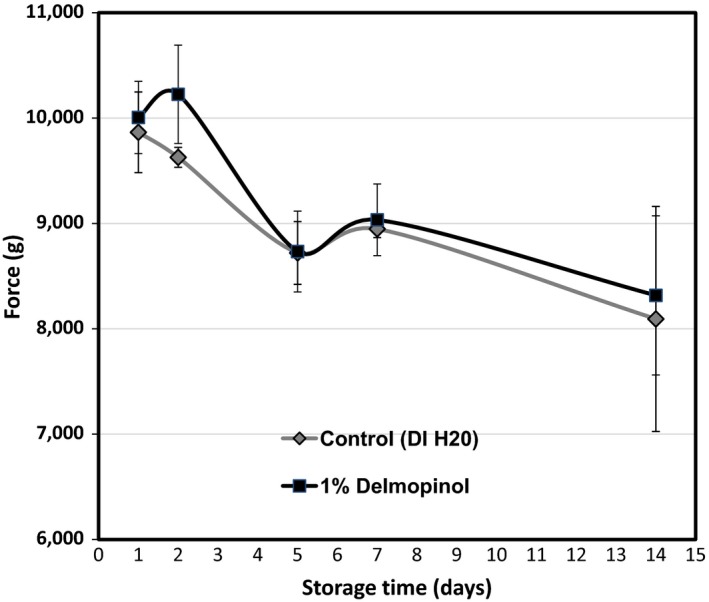
Skin hardness test (force (g) applied) on whole cantaloupes (“Athena”) after 0% or 1% delmopinol spray solution applications and 1, 2, 5, 7, and 14 days of storage at 4°C

After 7‐day storage, the hardness of skin samples of 1% delmopinol‐treated cantaloupes was not significantly different than control (DI water sprayed) samples (Figure 2). For the color measurements, no significant differences in L*, a*, b*, and ΔE* values, after 1, 2, 5, 7, and 14 days of refrigerated storage were found between 1% delmopinol‐treated cantaloupes and control cantaloupes (Table 3).

**Table 3:** Mean color measurements after spray application of 1% delmopinol on cantaloupe (“Athena”) during 14‐day storage at 4°C

	Day 1	Day 2	Day 5	Day 7	Day 14
Distilled water (Control)					
*L* mean	71.14 ± 1.68	77.52 ± 2.98	71.35 ± 2.34	70.67 ± 0.75	72.13 ± 2.50
*a* mean	−0.91 ± 0 .20	0.29 ± 0.97	−1.75 ± 0.51	−0.98 ± 1.24	−1.00 ± 0.80
*b* mean	19.63 ± 0.23	3.23 ± 0.83	18.60 ± 0.69	19.82 ± 1.25	19.53 ± 0.45
Δ*E*		17.40	0.94	1.24	0.83
1.0% Delmopinol					
*L* mean	70.67 ± 0.69	74.95 ± 2.10	70.23 ± 1.50	71.17 ± 1.59	70.96 ± 2.13
*a* mean	−0.50 ± 0.31	1.00 ± 0.84	−0.53 ± 0.43	−0.43 ± 0.73	−0.53 ± 0.81
*b* mean	19.59 ± 0.42	4.17 ± 0.92	19.99 ± 0.57	20.89 ± 0.45	20.37 ± 0.61
Δ*E*		16.08	0.59	1.39	0.83

*L *=* *0 yields black and *L *=* *100 indicates diffuse white; spectacular white; *a* = negative values indicate green, while positive values indicate magenta; *b* = negative values indicate blue and positive values indicate yellow; Δ*E* = Total color difference.

## 4 CONCLUSIONS

This research illustrates that a direct spray application with 1.0% delmopinol on the stem scar or on the skin of cantaloupes could reduce *Salmonella* cells by 3 log CFU/ml and make the bacteria more susceptible to sanitizers or physical removal. Furthermore, a direct spray application with 1.0% delmopinol resulted in no visible effect on color and on texture changes. This new approach of using an oral hygiene chemical, incorporated as an additional action to the regular cleaning and sanitizing program for netted surface fruits such as cantaloupes, could be an option to reduce human pathogens such as *Salmonella*. Furthermore, any new antimicrobial chemical used for this purpose should have no residual effects and not affect the visual appeal and texture qualities of the products. Postharvest food industrial applications of novel antimicrobial and surfactant chemicals, such as delmopinol hydrochloride, could be beneficial for reducing pathogenic bacteria on other raw foods.
